# Genomic diversity and thermal niches of *Aspergillus* molds disrupting rind formation of surface-ripened cheeses

**DOI:** 10.1128/aem.00032-26

**Published:** 2026-09-08

**Authors:** Umaojo Ojogbane, Christopher Tomo, Nicolas Louw, Benjamin E. Wolfe

**Affiliations:** 1 Department of Biology, Tufts University 1810, Medford, Massachusetts, USA; Chalmers tekniska hogskola AB, Gothenburg, Sweden

**Keywords:** temperature, spoilage, *Aspergillus*, fungi, cheese

## Abstract

Filamentous fungi play important roles in the development of surface-ripened cheese microbial communities and contribute to the aesthetics and flavors of these products. Much is known about the diversity and ecology of desirable cheese fungi, but our understanding of the natural history of cheese spoilage molds is limited. The goal of this work was to characterize the genomic diversity of *Aspergillus* species contaminating artisan cheeses and to identify how the abiotic environment of cheese (the substrate itself and temperature) may constrain the growth of *Aspergillus*. Comparative genomics identified two main species of *Aspergillus*, *A. westerdijkiae* and *A. ostianus*, as the spoilage molds across three different facilities in the Northeastern United States that experienced contamination events. Multiple genomic types of *A. westerdijkiae* were found across the different cheese production facilities, indicating that these contamination events are not caused by a single clonal strain. All *A. westerdijkiae* isolates produced ochratoxin A, but concentrations varied greatly across strains. RNA-sequencing of *A. westerdijkiae* on nutrient-rich lab media (malt extract agar) versus cheese curd agar identified a suite of pathways enriched in expression on cheese, including degradation of amino and fatty acids. Experiments measuring growth over a range of temperatures identified that spoilage *Aspergillus* species have a higher optimal growth temperature compared to desirable fungal species in cheese rinds and are outcompeted by *Penicillium* species at temperatures lower than 15°C. Global fungal metabarcoding databases suggest that *A. westerdijkiae* is not normally found in natural habitats of the Northeastern United States, and it may be introduced to this region.

**IMPORTANCE:**

Over the past decade, disruptive contamination events of *Aspergillus* spoilage molds have occurred at cheese production facilities in Massachusetts, Connecticut, and Vermont in the United States, causing aesthetic, flavor, and potential safety issues. Our work highlights independent introductions of different strains of *A. westerdijkiae* into multiple cheese facilities and suggests that temperature could be used to control the abundance of *Aspergillus* spoilage molds. Based on our analysis of the global distribution of *A. westerdijkiae,* it is not invading cheese facilities from local fungal populations and may be a contaminant in materials used for cheese production.

## INTRODUCTION

Fungal and bacterial communities have been used for hundreds of years to transform raw food materials into a diversity of fermented foods (1–4). One fermented food with a high abundance of fungi is surface-ripened cheese. These aged cheeses develop a microbial community on their surfaces that help provide unique appearances and textures and contribute flavors to the paste through decomposition of the milk components in the cheese curd (5–8). Depending on the specific style of cheese and production processes, a range of yeast and filamentous fungi—including *Penicillium*, *Scopulariopsis*, *Bifusarium*, *Debaryomyces*, *Sporendonema*, and *Chrysosporium*—can grow with bacterial species to form a multi-species community (5, 9, 10). Considerable past work has characterized the genomic and ecological diversity of these beneficial fungi on cheese rinds. These studies have shown how cheese rind fungi can interact with and adapt to cheese substrates (11–13), have strong interactions with bacterial species (9, 14–16), and can mediate microbial community assembly in cheese rinds (17–19). Many studies have also revealed how these fungi can determine the quality and safety of surface-ripened cheeses (5, 8, 20, 21).

Not all fungi that colonize the surface of a cheese are desirable, and unwanted spoilage molds can invade cheese rind microbial communities and potentially impact their appearance (5, 10). While we often hear reports from cheesemakers about unwanted filamentous fungi impacting their products (22) and some studies have noted the presence of undesirable molds on cheeses (23, 24), research on the natural history and basic ecology of spoilage molds in surface-ripened cheeses is surprisingly limited. This is especially true in the United States, where artisan cheese production is relatively new compared to Europe. Understanding what ecological processes control the growth of undesirable molds in cheeses can help provide “ecological levers” that cheesemakers can use to manage fungal communities in their products.

**Fig 1:**
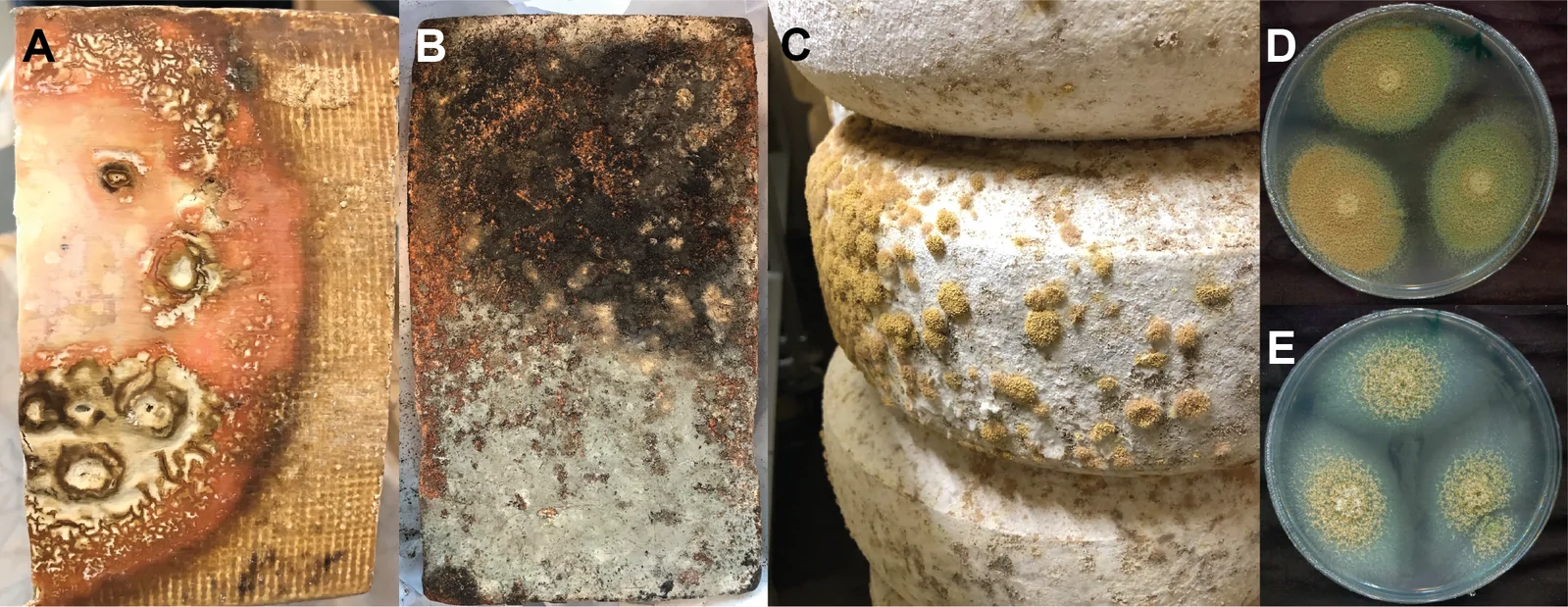
Phenotypes of *Aspergillus* spoilage molds from surface-ripened cheeses produced in the United States. (**A–C**) Various phenotypic presentations of spoilage *Aspergillus* species on cheese rinds. In panel **A**, large white and black patches appeared where gaps in rinds formed on a washed-rind cheese. In panel **B**, black dusty patches appeared on a block of cheddar. In panel **C**, yellow patches that were raised up off the rind surface appeared on a natural-rind cheese. (**D and E**) Morphological appearance of representative *Aspergillus* growing on malt extract agar. Panel **D** is *A. ostianus*, and panel **E** is *A. westerdijkiae*.

Recently, our laboratory has been contacted by cheesemakers on several occasions regarding the growth of abnormal fungi causing wrinkling and discoloration of surface-ripened cheese rinds (Fig. 1). Preliminary microscopy and single-locus (internal transcribed spacer, or ITS) sequencing putatively identified these fungi as *Aspergillus* species in section *Circumdati. Aspergillus* species have been previously detected in cheeses available to consumers (9). The undesirable appearance of both natural and washed-rind cheeses contaminated with these two species, including unusual black pigmentation or loss of normal rind discoloration, can cause challenges for cheese producers. Beyond product aesthetics, *Aspergillus* species in section *Circumdati* are well-characterized producers of ochratoxins and other mycotoxins (25). These secondary metabolites can cause harm at both microbial and human scales, and many regulatory agencies screen food products for ochratoxins and aflatoxins due to safety concerns (20, 26). Recent work by our lab and collaborators has demonstrated that *A. westerdijkiae* can strongly inhibit beneficial bacteria and fungi in cheese rind communities and produces a suite of antibacterial metabolites, including penicillic acid, that can have antimicrobial activity (27). The genomic identities and ecology of these molds have not been characterized, but this information is needed to help diagnose and manage contamination events of *Aspergillus* in cheese facilities.

*A. westerdijkiae* and other *Aspergillus* species in section *Circumdati* are capable of growing in diverse conditions and have been isolated from environments, including marine sediments (28, 29), indoor air and surface samples (25, 30, 31), and coffee beans (32, 33). *A. westerdijkiae* has been reported on a variety of foods, including pistachios, rice, grapes, fermented sausages, and cheese (23, 31, 34–36). Based on searching the literature for sequence-based identifications of the fungus, we are unaware of previously documented food contamination or isolation of *A. westerdijkiae* in the Northeastern United States. Additionally, the global distribution and native range of *A. westerdijkiae* are not known. Various studies encounter this fungus in food and agricultural systems, the built environment, and sometimes in natural ecosystems (29, 31, 37–39), but a systematic survey to understand its putative distribution across the globe has not been conducted. Understanding where in the world and in what habitats *A. westerdijkiae* resides could help us better predict when it may cause problems in food production environments.

In this study, we employ a variety of approaches to characterize the diversity and ecology of *Aspergillus* strains isolated from surface-ripened cheeses made and aged in the Northeastern United States. We first used phylogenomics to determine the genomic diversity of multiple *Aspergillus* strains isolated from cheeses. Because of the recent expansion of this fungus on cheese in the Northeastern United States and because *Aspergillus* species are not typically found on cheeses, we predicted that a single cheese-adapted strain of *A. westerdijkiae* may be spreading throughout cheese caves. To better understand how a fungus that often grows on plant substrates might interact with the environment and resources in a cheese, we used RNA-sequencing (RNA-seq) to identify differentially expressed genes on protein-rich cheese curd agar (CCA) compared to a standard lab medium rich in carbohydrates (malt extract agar, or MEA). Given the nutritional constraints that limit many microbes from establishing growth on cheese (40, 41), we predicted that CCA would elicit a large transcriptomic response, particularly related to scarce resources like free amino acids and iron (42, 43). We also assessed the thermal niche of *A. westerdijkiae* on cheese relative to other cheese filamentous fungi, including *Penicillium* species that are commonly found on cheese rinds. Because *Aspergillus* species generally prefer warmer temperatures compared to *Penicillium* species, we predicted that the optimal growth ranges of our *Aspergillus* isolates on cheese would be higher than those of *Penicillium* and other cheese fungi. Finally, to explore the potential global distribution of *A. westerdijkiae,* data from GlobalFungi.com were used. Because this fungus has only recently appeared in Northeastern United States cheese facilities, we predicted that this fungus does not naturally occur in soils or other environments in this region and that it may be introduced from other parts of the world.

## MATERIALS AND METHODS

### Isolation of *Aspergillus* cultures and genomic DNA

To isolate *Aspergillus* species from cheese rinds, we used a serial dilution approach on plate count agar with milk and salt (PCAMS) as previously described (44). Cheeses with putative *Aspergillus* were provided to our lab when cheesemakers were having rind development or aesthetic issues. The first *Aspergillus* contamination event was in 2012 from a goat milk cheese with a natural rind (resulting in strain Aw2012). The rind of this cheese had a dark black flaking appearance. Later in 2019, a larger contamination event occurred at another facility, with both washed-rind (Fig. 1A) and natural-rind (Fig. 1B) cheeses affected. On washed-rind cheeses, the main macroscopic features of *Aspergillus* were spots on the rind where the typical orange rind pigmentation was lost and white or black-gray spots appeared (Fig. 1A). On natural rinds such as block cheddars, dark black dusty areas dominated where *Aspergillus* was present (Fig. 1B). Isolation of putative *Aspergillus* from this facility resulted in strain Aw2019. Another *Aspergillus* population expansion happened in a facility in 2023, with striking yellow patches that were dusty and raised off the surface of natural-rind cheeses (Fig. 1C). Because there were several unique macroscopic phenotypes on these cheeses, multiple isolations resulted in several strains from this facility, including strains Aw2023_1, Aw2023_3, and Aw2023_5.

We isolated putative *Aspergillus* species from mixed culture plates resulting from serial dilutions of rind samples plated on PCAMS with 50 mg/L of chloramphenicol as previously described (44). We considered putative *Aspergillus* colonies to be those that were more raised on plates compared to typical cheese rind *Penicillium* colonies and with yellow conidia. To confirm that the putative isolates were *Aspergillus* species, we observed conidiophores at 200× magnification under a light microscope. Pure cultures were generated by streaking putative *Aspergillus* species from mixed culture plates using a sterile wooden dowel. All plates for isolations were incubated at 24°C in the dark for 7 days. We only sequenced a few *Aspergillus* isolates associated with contaminated cheese at each site because we were largely interested in understanding the species causing the issue, not the population structure within a specific contamination event. We recognize that this ignores potential variation within each fungal population and that additional sampling would be needed to clearly define the population structure of this fungus.

To obtain DNA for whole-genome sequencing, we scraped approximately 50 mg of spores and mycelium from a pure culture using a wooden dowel and placed it into 100 µL of molecular-grade water. We then used the Qiagen DNeasy PowerSoil Pro Kit according to the manufacturer’s instructions to extract genomic DNA. Genomic libraries were then prepared with either in-house library preparation kits for strains *A. westerdijkiae* Aw2019 and *A. westerdijkiae* Aw2012 (NEBNext Ultra DNA Library Prep Kit for Illumina with 2 × 150 bp paired-end sequencing on an Illumina NextSeq 550 at the Tufts University Core Facility) or using a sequencing pipeline at SeqCoast for strains Aw2023_1, Aw2023_3, and Aw2023_5 (Illumina DNA Prep tagmentation kit with unique dual indexes and 2 × 150 bp paired-end sequencing on the Illumina NextSeq2000).

### Genome assembly, annotation, and comparative genomics

To determine how the cheese *Aspergillus* isolates were related to previously characterized *Aspergillus* species, we constructed a whole-genome phylogeny. Genomes were assembled using SPAdes v3.15.3 and annotated using AUGUSTUS v3.2.2 (45) using *Aspergillus fumigatus* as the “species” parameter. Ten genomes of *Aspergillus* species in section *Circumdati* of *Aspergillus* were downloaded from NCBI databases (accessed on 10 August 2024; Table S1). To explore the phylogenomic relationships among isolated and publicly available *Aspergillus* strains, we used Orthofinder v2.3.8 (46) to screen amino acid sequences for single-copy orthologous genes. Specifically, to cluster amino acid sequences based on similarity, we used the DIAMOND v2.0.13.151 (47) algorithm. The resulting 6,885 single-copy orthologous amino acid sequences shared among the set of *Aspergillus* species were aligned with MAFFT v7.402 using the default parameters. Of these, we generated a single concatenated multiple sequence alignment, which we used as an input to generate a single species tree following a maximum likelihood-based approach with IQ-TREE V2.3.0 (48). To select the best-fitting model, we used ModelFinder (49).

To identify putative biosynthetic gene clusters (BGCs), we screened the assembled draft genomes of all five isolates for BGCs using antiSMASH (v.8.0.2) with detection strictness set to relaxed (50). To investigate specific clusters of interest, we performed BLASTp searches against a reference genome in Geneious and visualized gene cluster similarities using Clinker (51).

To assess whether cheese-associated *A. westerdijkiae* strains (Aw2012, Aw2019, and Aw2023_1) possess unique genes that may enable them to thrive in the cheese environment, we used CD-HIT v4.8.1 (52) to cluster all predicted protein sequences from all seven *A. westerdijkiae* strains at an 85% amino acid identity threshold to limit the splitting of closely related homologs while retaining functionally distinct gene families. Genes were defined as “unique” if they appeared in only one strain. Functional enrichment of these unique genes was performed using KOBAS-i v3.0 (53) to determine whether any strain-specific genes corresponded to specific biological pathways.

### Ochratoxin A quantification

To assess the ochratoxin A (OTA) production potential of the five *Aspergillus* isolates (Aw2012, Aw2019, Aw2023_1, Aw2023_3, Aw2023_5), 10 µL of a 200 CFU/µL spore suspension was spot-plated onto 10% cheese curd agar plates. Each strain was plated in three biological replicates. The plates were incubated for 14 days at 15°C to mimic typical cheese cave conditions. Following incubation, the entire agar medium from each replicate plate was harvested, transferred into Whirl-Pak bags, and stored at −20°C until downstream ochratoxin analysis.

OTA was quantified using liquid chromatography–tandem mass spectrometry (LC–MS/MS) at Trilogy Analytical Laboratory (Washington, MO, USA). OTA was extracted from the samples using a 3:1 (vol/vol) methanol:water solution. Samples were vortexed for 3 min, shaken for 1 h, and centrifuged at 3,000 rpm for 5 min. The resulting supernatants were purified using solid-phase cleanup columns (QP1100; Trilogy Analytical Laboratory), diluted in 2% acetic acid, and transferred to autosampler vials for analysis. Samples exceeding the calibration range were diluted and reanalyzed.

A Shimadzu high-performance liquid chromatography system equipped with an SIL-40 Series autosampler was used, and 5 µL of sample was injected onto a Phenomenex Kinetex column maintained at 40°C at a flow rate of 0.5 mL/min. Mobile phase A consisted of water with 0.1% formic acid and 4 mM ammonium formate, and mobile phase B consisted of methanol with 0.1% formic acid and 4 mM ammonium formate, delivered at a flow rate of 0.5 mL/min. A gradient elution program was used, starting at 5% B (held for 1 min), increasing to 50% B over 6 min, held for 12 min, then returning to 5% B over 4 min, followed by a 4-min re-equilibration period.

Mass spectrometric detection was performed in positive electrospray ionization mode with the following parameters: interface temperature 350°C, ion spray voltage 1,700 V, curtain gas 40 psi, collision gas 9 psi, and ion source gases set at 40 psi (gas 1) and 60 psi (gas 2). OTA was detected using scheduled multiple reaction monitoring, monitoring the two most intense precursor–product ion transitions within a 1.2-min retention window. Dwell times were automatically optimized using SCIEX OS 3.0 (Framingham, MA, USA). Quantification was performed using an OTA standard curve generated from an analytical standard (TSL-503; Trilogy Analytical Laboratory).

### RNA-sequencing

To determine what genes or pathways may be important for *A. westerdijkiae* growth on cheese curd, we used RNA-seq to identify differentially expressed genes on a cheese-like medium (CCA) compared to a standard carbohydrate-rich medium (MEA). We had a range of standard fungal media types that we could have used as a comparison to CCA, including PDA. We decided to use MEA for the RNA-seq experiment because it has a high amount of both carbon and nitrogen. Experimental stocks (15% glycerol in 1× phosphate-buffered saline, or PBS) of *A. westerdijkiae* stored at −80°C were thawed, diluted to a standardized concentration, and inoculated onto 100 mm Petri dishes containing 20 mL of either MEA (30 g/L malt extract, 15 g/L agar, and pH-adjusted to 5.5 in distilled water) or CCA (10% lyophilized cheese curd, 0.5% xanthan gum, 1.7% agar, and 3% NaCl). All replicates (*n* = 3) were incubated in the dark at 24°C for 5 days prior to RNA extraction.

After incubation, fungal cells were harvested by scraping biomass from the surface of the solid media using sterilized razor blades and frozen at −80°C submersed in Qiagen RNAlater Stabilization Solution. After at least 24 h at −80°C, cells were thawed and removed from the solution, frozen in liquid nitrogen, and manually ground to a fine powder using a pre-sterilized and pre-cooled mortar and pestle. Up to 200 mg of this powder per sample was then processed using the Qiagen RNeasy Plant Mini Kit. This protocol was completed according to the manufacturer’s instructions with one alteration: the column was incubated with 80 μL of Qiagen RNase-free DNase for 15 min at room temperature between the two RW1 wash steps. Each of the three biological replicates per treatment was processed separately.

Fungal mRNA was isolated using the New England Biolabs NEBNext Poly(A) mRNA Magnetic Isolation Module according to the manufacturer’s instructions. Isolated mRNA was then library prepped for Illumina sequencing with the NEBNext Ultra II RNA Library Prep Kit for Illumina following the manufacturer’s instructions for preparing intact RNA. Prepped mRNA was pooled and sequenced on an Illumina NextSeq 550 (single-end sequencing, 150 cycles) at the Tufts University Core Facility.

Raw Illumina reads were mapped to an assembled *A. westerdijkiae* strain Aw2019 genome using Geneious Prime mapper (version 2023.1.2) at medium–low sensitivity, with reads mapping to multiple best matches randomly assigned. Expression levels of each sample were calculated using the Geneious Prime calculate expression levels tool, with ambiguously mapped reads counted as partial matches. Differential expression was calculated using the DESeq2 plugin (54). Genes were considered differentially expressed if *A. westerdijkiae* expression when grown on CCA alone was greater than doubled (log_2_ ratio >1) or less than halved (log2 ratio < −1) compared to growth on MEA alone at a corrected *P*-value < 0.05, adjusted for false discovery rate (FDR) using the Benjamini–Hochberg procedure (54).

The KOBAS-i (version 3.0) gene-list enrichment tool (53) was used to identify known metabolic pathways from the Kyoto Encyclopedia of Genes and Genomes (KEGG) database (55) that were enriched with differentially expressed genes. The *A. fumigatus* genome was selected as the background genome to test for enrichment due to its extensive annotation, close phylogenetic relationship to *A. westerdijkiae*, and the lack of a fully annotated *A. westerdijkiae* genome within the KOBAS-i database.

### Thermal niche experiments

To determine how temperature may control the growth of spoilage *Aspergillus* species compared to typical cheese fungi, 18 fungal strains were selected for thermal niche screens: five strains of *Aspergillus* (designated Aw2023_1, Aw2023_3, Aw2023_5, Aw2012, and Aw2019), ten strains of *Penicillium* (designated SAM, 162_3FA, #12, 258, 259, 261, 277, 280, 285, and 288), and three other typical cheese molds (*Bifusarium domesticum* strain 554A, *Geotrichum candidum* strain 242A, and *Scopulariopsis* sp. strain 165_5). SAM is *P. camemberti*, a commercial starter culture: 162_3FA is *P. biforme* (12), #12 is *P. solitum* (9, 12, 56, 57), and the remaining strains (258, 259, 261, 277, 280, 285, and 288) represent various *Penicillium* species isolated from cheese rinds as previously described (58). All strains were stored at −80°C in 15% glycerol in 1× PBS prior to use in experiments. CFUs of experimental glycerol stocks were determined by serially diluting on PCAMS as previously described (44).

To determine mycelial growth rate at a range of temperatures, the strains were cultured on potato dextrose agar (PDA). We selected this medium because PDA is commonly used for temperature growth assays of *Penicillium* and *Aspergillus* species. We acknowledge that fungal growth rates will be different on actual cheese curds, but PDA provides a common medium used by researchers around the world for standardized growth assays. Each strain was inoculated onto the center of a 100 mm Petri dish containing 20 mL of medium at a concentration of 200 CFUs/μL, with 10 μL of the inoculum spot-plated onto each plate, with five replicates for each medium type and temperature that were all set up on the same day. While separate experimental replicates on separate days would have been ideal, it would have expanded the scope of the experiment beyond the resources we had for this work. In our experience, these temperature growth assays are robust across different experimentalists and days.

The experiment was conducted across a temperature range of 10°C–35°C, with the cultures incubated at 5°C intervals (10°C, 15°C, 20°C, 25°C, 30°C, and 35°C) in temperature-controlled incubators in the dark. Growth was monitored over 15 days, with measurements taken on days 3, 5, 7, 9, 11, 13, and 15. At each time point, the diameter of each fungal colony was measured in millimeters using a caliper. To confirm that the patterns of growth across temperatures that we observed for *A. westerdijkiae* on PDA were similar in a cheese-like environment, we used the same experimental setup as the above experiments, but replaced PDA with CCA and only used a single strain (Aw2019). We also added an additional lower temperature (5°C) to confirm the lowest temperature at which *A. westerdijkiae* can grow in a simulated cheese environment.

Growth rate (mm d⁻¹) was estimated as the slope of a linear regression of colony diameter (mm) over time (days) at each temperature (59, 60). To test whether there were differences in growth at higher temperatures, we compared mean growth rates across all strains at 30°C using a one-way analysis of variance (ANOVA) to test for differences among individual species. To test for broad differences in growth rates across temperatures, we grouped strains into *Aspergillus* (*n* = 5) and other cheese-associated fungi (*n* = 13) and performed a one-way ANOVA.

To identify the lower temperature threshold for growth of *A. westerdijkiae*, we conducted a fine-scale temperature experiment with strain Aw2019 across 1°C increments. Cultures were grown on PDA in 60 mm Petri dishes and inoculated as described above for the mycelial growth rate assay. The inoculated plates were incubated at 9°C, 10°C, 11°C, 12°C, 13°C, or 14°C for 15 days, with five replicates per temperature.

Following incubation, the entire agar plate and fungal biomass from each replicate were harvested and transferred into a sterile Whirl-Pak bag. We added 10 mL of sterile 15% glycerol in 1× PBS to each bag, and the contents were thoroughly homogenized. A 100 μL aliquot of the homogenate was spread onto PDA plates and incubated at 25°C for 7 days. CFUs were quantified and reported as the mean for each temperature.

### *Aspergillus*–*Penicillium* competition experiments

To test how *A. westerdijkiae* might compete with a beneficial cheese rind fungus across a range of temperatures in a simulated cheese environment, we co-cultured *A. westerdijkiae* strain Aw2019 with *P. biforme* strain 162_3FA. Both of these strains were isolated from natural-rind cheeses from the same cheese facility. *P. biforme* strain 162_3FA has been previously described and characterized (12). To inoculate the strains, they were thawed from frozen glycerol stocks with a known concentration of CFUs and diluted to a standardized concentration of 100 CFUs/μL. For each biological replicate (*n* = 5), 10 μL of inoculum with a 1:1 CFU ratio of both fungi was inoculated onto 150 μL of CCA in a 1.5 mL microcentrifuge tube as previously described (44). Tubes were incubated in the dark at either 10°C, 15°C, 20°C, 25°C, 30°C, or 35°C in a microcentrifuge tube rack. Their lids were open and covered with sterile AeraSeal film (Millipore Sigma, Burlington, MA, USA) to prevent environmental and cross-experiment contamination while allowing gas exchange. To help maintain humidity, ten 1.5 mL microcentrifuge tubes containing 1 mL each of deionized water were also placed throughout the tube rack that was covered and placed inside a sealed bag while it was incubated. The placement of each tube within the rack was randomized to account for spatial variations in moisture and temperature within each incubator.

After 7 days of incubation, 500 μL of 1× PBS was added to each microcentrifuge tube, and its contents (cells, CCA, and PBS) were pestled using a sterile micropestle until homogeneous. The contents of each tube were serially diluted, plated onto PCAMS, and incubated at either 10°C or 30°C (to favor the growth of *P. biforme* and *A. westerdijkiae*, respectively) for colony counting. To test whether competitive outcomes between *A. westerdijkiae* and *P. biforme* varied across temperature, we performed a two-way ANOVA on fungal abundance (log₁₀ CFU mL⁻¹ +1) with species identity and temperature as fixed effects. Tukey’s honestly significant difference was used to identify differences in competitive outcomes between species at each temperature.

### Global distribution of *A. westerdijkiae* based on GlobalFungi

To determine whether *A. westerdijkiae* is normally present in the Northeastern United States or whether it may be introduced to cheese facilities in this region, we used the global fungal database GlobalFungi (61). This online database compiles fungal biodiversity studies that use ITS metabarcoding to characterize fungal communities in a range of habitats. It includes data from ITS metabarcoding studies of 84,972 samples from 846 studies spanning all continents. We accessed the data that included ITS1 or ITS2 reads assigned to *A. westerdijkiae* species hypotheses in the UNITE database (https://unite.ut.ee/) in August 2025. We filtered the resulting 739 samples to exclude studies where only a single read of *A. westerdijkiae* was reported or where *A. westerdijkiae* reads were less than 0.01% of the total ITS reads in a sample. We then used the reported latitude and longitude for each of the remaining 320 samples to create a map of the global distribution of ITS metabarcoding studies that have detected putative *A. westerdijkiae*. To ensure that any lack of detection of *A. westerdijkiae* was not due to a lack of studies/samples in the GlobalFungi database from the Northeastern United States, we also made a map of all samples in GlobalFungi from this region.

## RESULTS

### Comparative genomics reveals distinct strains of *Aspergillus westerdijkiae* as well as *Aspergillus ostianus* as contaminants of Northeastern United States cheese rinds

Comparative genomics revealed two key results regarding the genomic diversity of the *Aspergillus* strains isolated from these Northeastern United States cheeses: (i) there are two distinct species that can colonize cheese rinds, *A. westerdijkiae* and *A. ostianus*, and (ii) there are several distinct strains of *A. westerdijkiae* that have invaded different facilities.

**Fig 2:**
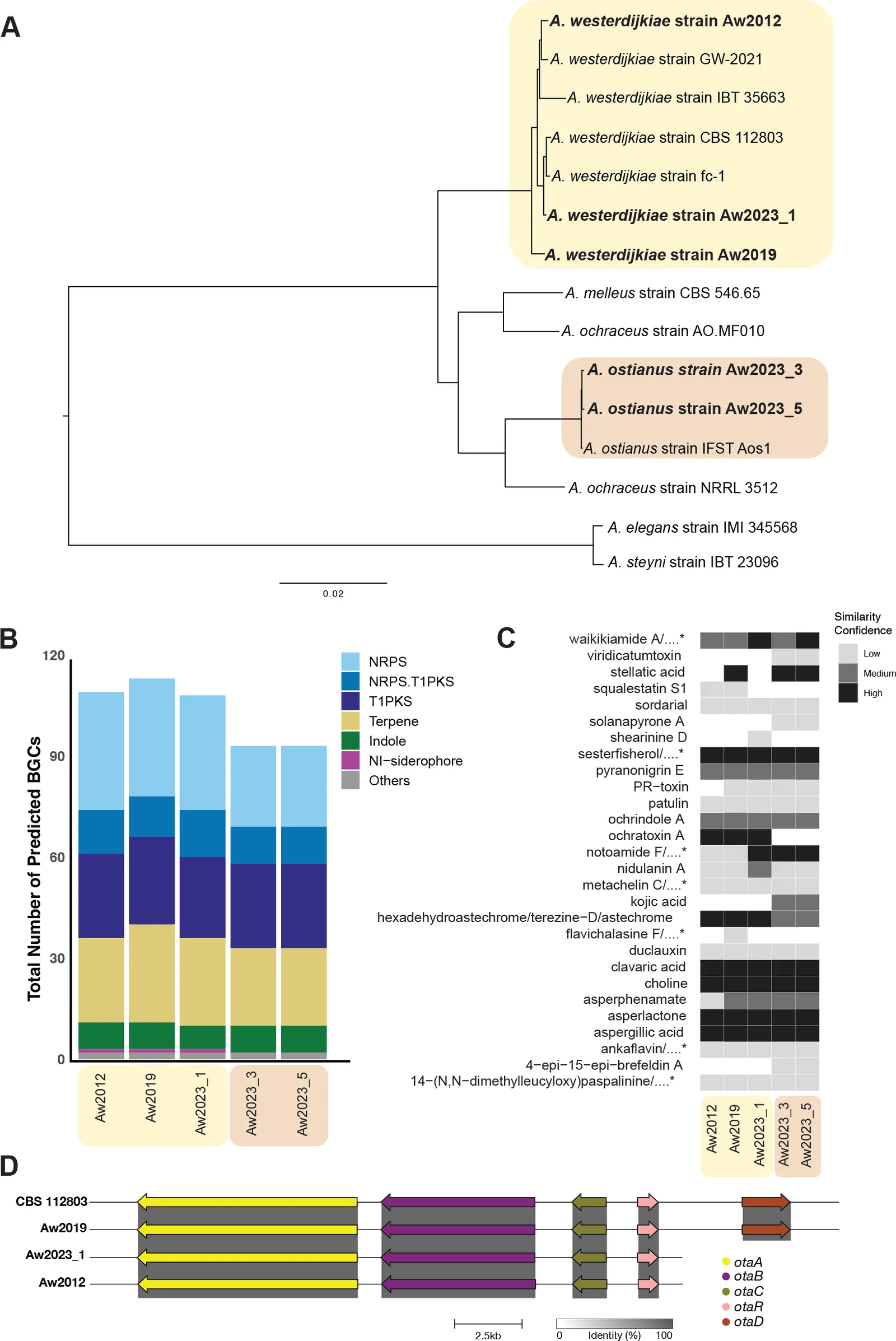
Phylogenomic and biosynthetic gene cluster analyses of spoilage *Aspergillus* species from cheese rinds. (**A**) Maximum likelihood-inferred phylogeny of *A. westerdijkiae*, *A. ostianus*, and closely related species. Strains that were isolated from cheese for this study are bolded, and parts of the tree containing these strains are highlighted in yellow (for *A. westerdijkiae*) and orange (for *A. ostianus*). This tree was constructed with a concatenated alignment of 6,885 single-copy orthologous sequences identified by Orthofinder. Protein sequences were aligned with MAFFT, and tree construction was performed with IQ-TREE using the JTT+F+R10 model and 1,000 ultrafast bootstrap replicates. All internal branches had 100% bootstrap support, except for the branch leading to *A. ostianus* Aw2023_5, which had 82% bootstrap support. (**B**) Total number of predicted BGCs identified by antiSMASH for each genome. BGC classes include NRPS, NRPS-T1PKS hybrids, T1PKS, terpenes, indoles, and other cluster types. Bars are color-coded by BGC category. (**C**) Heatmap showing presence and similarity of known secondary metabolite pathways across strains. Shading intensity corresponds to cluster similarity. When an asterisk (*) is present for putative BGCs, the name was truncated for visual clarity. See Table S3 for full names. (**D**) Synteny comparison of the OTA biosynthetic gene cluster across representative *A. westerdijkiae* strains.

Two isolates (Aw2023_3 and Aw2023_5), which initially had fairly distinct appearances as colonies with different surface textures and slightly different colors, were very closely clustered with one of the only other genomes of *A. ostianus* available, strain IFST Aos1 (Fig. 2A). Aw2023_5 formed white to pale, radially striated colonies with mostly submerged (flat) mycelium, while Aw2023_3 developed yellow-ochre pigmented conidia with aerial mycelium. Sporulation was more evident in Aw2023_3 with conidial heads, whereas Aw2023_5 sporulated sparsely. Sclerotia were absent in both isolates under the tested conditions. These isolates had more dense conidiophore production across their colonies and were often a more intense yellow color in culture compared to *A. westerdijkiae* (Fig. 1D and E). *A. ostianus* and *A. ochraceus* are the most closely related species of *Aspergillus* to *A. westerdijkiae* and can have similar colony appearances (62). To the best of our knowledge, there is only one other report of *A. ostianus* on cheese from Brazil (63).

Within the cluster of *A. westerdijkiae* genomes, cheese isolates did not cluster together (Fig. 2A), demonstrating that at least three distinct strains of this species have been introduced into cheese aging facilities in the Northeastern region of the United States. The strain *A. westerdijkiae* Aw2019 is highly divergent compared to *A. westerdijkiae* strains and is on a branch by itself. The two other *A. westerdijkiae* strains that we isolated (Aw2012 and Aw2023_1) clustered with other strains isolated from soil and plant environments. *A. westerdijkiae* strain Aw2023_1 was isolated from the same cheese sample as *A. ostianus* strains Aw2023_3 and Aw2023_5, demonstrating that two *Aspergillus* species can invade and impact cheeses at the same time.

To determine the presence and function of strain-specific genes among *A. westerdijkiae* isolates, we constructed a protein-based pangenome. Overall, the pangenome comprised 13,321 protein gene families, of which 8,633 were core clusters shared across all strains, 2,680 were accessory clusters present in 2–6 strains, and 2,008 were unique clusters present in a single strain. Strain-specific gene counts varied across isolates. Strain IBT 35663 contained the largest number of unique clusters (694), followed by Aw2019 (478), GW-2021 (336), Aw2012 (165), CBS 112803 (159), fc-1 (102), and Aw2023_1 (74) (Table S2). Despite the presence of these strain-specific gene families, functional pathway enrichment analysis indicated that unique genes did not map to specific metabolic or functional pathways. Instead, strain-specific genes consistently mapped to the same broad KEGG pathways identified across all strains, including amino acid metabolism, carbohydrate metabolism, lipid metabolism, secondary metabolite biosynthesis, and core cellular processes (Table S2).

We assessed the genomic potential for secondary metabolite production in the five cheese-associated *Aspergillus* strains using the fungal version of antiSMASH. The class composition was conserved across genomes, with nonribosomal peptide synthetase (NRPS) clusters as the most abundant, followed by type I polyketide synthase (T1PKS) and terpene clusters (Fig. 2B; Table S3). Hybrid clusters such as NRPS.T1PKS were found in all genomes. We identified 471 BGCs, ranging from 84 to 103 per genome. Of these, 117 BGCs (24.8%) showed similarities with clusters known to produce secondary metabolites (Fig. 2C), while 354 (75.2%) were uncharacterized. *A. westerdijkiae* isolates generally carried more BGCs (Aw2019 highest with 103 BGCs) (Fig. 2B). Per genome totals were higher than earlier reports of 88 BGCs for *A. westerdijkiae* (64), which may reflect strain-specific expansions and the inclusion of partial or putative clusters in newer antiSMASH versions (50).

Several BGCs were shared across most genomes, including aspergillic acid, asperphenamate, clavaric acid, duclauxin, and choline. Species-specific differences were also evident. All three *A. westerdijkiae* genomes contained a putative OTA biosynthetic gene cluster, whereas the OTA cluster was not detected in the *A. ostianus* genomes. OTA is a mycotoxin commonly produced by several *Aspergillus* and *Penicillium* species (65). The core biosynthetic genes (*otaA*, *otaB*, *otaC*, *otaR*, and *otaD*) (65) were present in only strain Aw2019, while Aw2012 and Aw2023_1 lacked the *otaD* gene (Fig. 2D). The *otaD* gene encodes a halogenase that catalyzes the chlorination step in the ochratoxin pathway, converting ochratoxin B (OTB) into OTA (65). Both *A. ostianus* isolates (Aw2023_3 and Aw2023_5) lacked an intact OTA cluster. The absence of a complete OTA cluster in *A. ostianus* is consistent with previous reports describing truncation or absence of this cluster in comparison with other species in section *Circumdati* (66).

To corroborate the genomic predictions of OTA production across the *Aspergillus* strains and species, we measured OTA production of all three *A. westerdijkiae* strains (Aw2019, Aw2012, and Aw2023_1) and the two *A. ostianus* isolates (Aw2023_3 and Aw2023_5). OTA production varied between strains, with Aw2012 producing higher mean levels of OTA (mean 54,454.7 µg/kg, SD = 7194.4) than Aw2023_1 (mean 3,775.7 µg/kg, SD = 368.5) and the lowest levels in Aw2019 (mean 16.7 µg/kg, SD = 3.0). OTA levels were below reporting limits (<1 µg/kg) in the two *A. ostianus* isolates Aw2023_3 and Aw2023_5. These results agree with earlier reports of variable OTA production in *A. westerdijkiae* strains (67) and inconsistent detection of OTA in *A. ostianus* isolates (25).

*Aspergillus ostianus* isolates carried putative BGCs that were unique to this species, including kojic acid, 4-epi-15-epi-brefeldin A, and viridicatumtoxin. Viridicatumtoxin is a fungus-derived tetracycline-like polyketide antibiotic whose gene cluster has been characterized in *P. aethiopicum* (68, 69), and related pathways occur in some *Aspergillus* species (70). Its ecological role on cheese rinds is not known, but as a broad-spectrum inhibitor, it may mediate competitive interactions with bacteria.

### The cheese rind substrate induces broad transcriptomic changes in *A. westerdijkiae*

**Fig 3:**
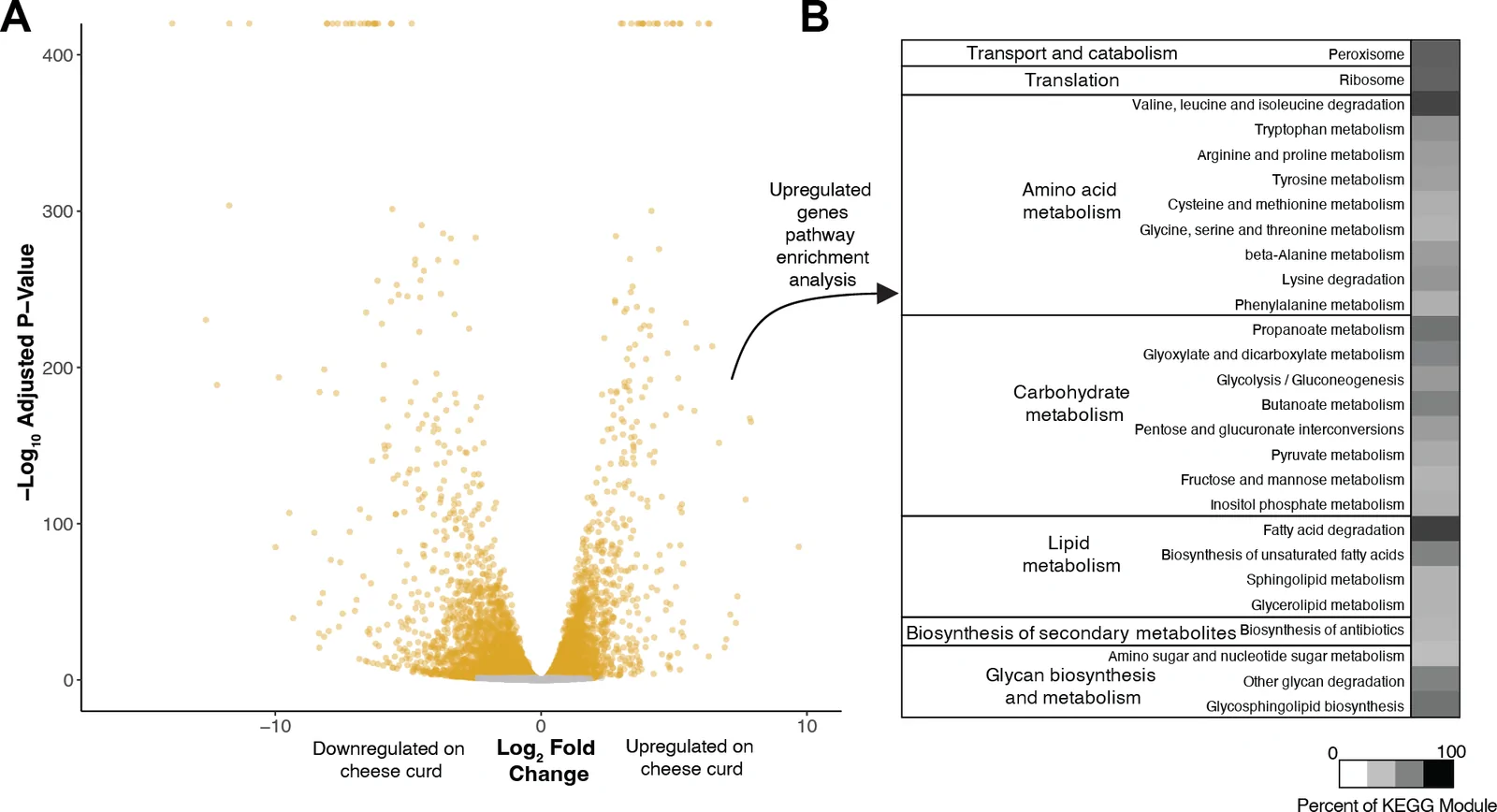
Growth on cheese curd induces strong transcriptomic responses in *A. westerdijkiae*. (**A**) Volcano plot of RNA-seq data from *A. westerdijkiae* grown on malt extract agar versus cheese curd agar. Each dot represents a transcript in the *A. westerdijkiae* strain Aw2019 genome. Dots in a horizontal line along the top of the graph represent genes with very high −log_10_ adjusted *P*-values (highly significant) and would have been too high on the graph to be effectively visualized. (**B**) Distribution of KEGG pathways that were significantly enriched in the set of genes upregulated on cheese curd agar, based on KOBAS-i pathway enrichment analysis. Shading indicates the % of total genes in a KEGG module that were included in the upregulated gene set.

To understand what genes and metabolic pathways may be important for *A. westerdijkiae* to grow on cheese, we compared the transcriptome of *A. westerdijkiae* strain Aw2019 grown on MEA versus CCA. MEA is a high-nutrient standard laboratory medium rich in carbohydrates, while CCA mimics the substrate available for fungi when growing in a cheese cave. CCA has many resources available for microbial growth, but has low concentrations of easily accessible carbohydrates, and access to resources is mediated by decomposition of proteins and fats (6, 71). Differential expression analysis between *A. westerdijkiae* grown on the two substrates revealed that growth on CCA led to significant changes in gene expression (Fig. 3A): 2,359 genes were identified as significantly downregulated on CCA compared to MEA (~21% of predicted protein-coding genes), and 1,299 genes were identified as significantly upregulated (11.6% of predicted protein-coding genes) (Table S4).

Pathway enrichment analysis identified 36 significantly enriched KEGG pathways in the genes upregulated on CCA (Table S5). There were no pathways that were significantly enriched in the downregulated genes (Table S5). The predicted functions of KEGG pathways in the significantly upregulated genes suggest very strong shifts in the metabolic strategies of *A. westerdijkiae* when grown on cheese (Fig. 3B).

Two KEGG modules that had the highest enrichment, with nearly 75% of genes in the pathway represented in the upregulated genes, were valine, leucine, and isoleucine degradation and fatty acid degradation (Fig. 3B). Cheese consists of a large amount of amino acids bound in casein as well as various fats that fungi can degrade into fatty acids for metabolism, and these pathways are commonly enriched in transcriptomic studies of bacteria and fungi in the cheese rind system (71, 72). A variety of other amino acid, carbohydrate, and lipid metabolism pathways were also upregulated by *A. westerdijkiae* when grown on cheese curd agar (Fig. 3B).

### Spoilage *Aspergillus* species have a higher optimal growth temperature compared to other cheese rind fungi

**Fig 4:**
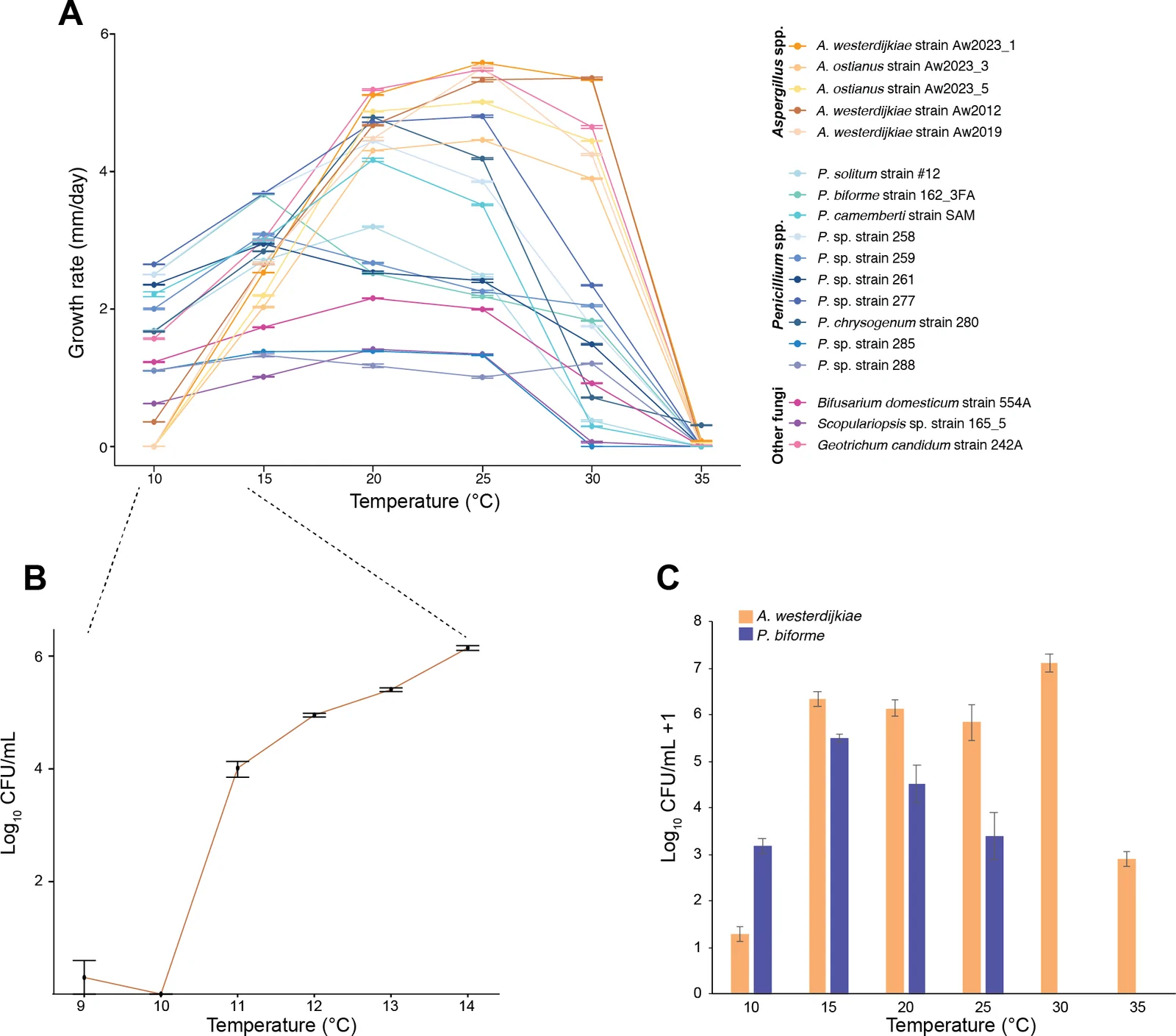
Growth and competition of *A. westerdijkiae* across a range of temperatures. (**A**) Changes in radial growth rate across different incubation temperatures on PDA. *Aspergillus* (shades of yellow), *Penicillium* (shades of blue), and other genera (shades of pink). Error bars show the standard error of the mean. Similar patterns of growth across the same range of temperatures were observed on cheese curd agar (Fig. S1). (**B**) Abundance of mean CFUs/mL of *A. westerdijkiae* strain Aw2019 between 9°C and 14°C; the dashed line in panel A marks the low-temperature region highlighted here. Error bars show one standard error of the mean. (**C**) Growth of *A. westerdijkiae* and *P. biforme* in pairwise co-culture across a range of temperatures. Bars represent the mean of five replicates, and error bars are one standard deviation of the mean.

To determine how temperature can influence the growth of cheese rind fungi, mycelium diameter was measured across a range of 6 temperatures (10°C, 15°C, 20°C, 25°C, 30°C, and 35°C) for isolates of *Aspergillus, Penicillium,* and other cheese-associated molds on PDA (Fig. 4A). Growth rates varied markedly across the thermal gradient, with clear interspecific differences. At 30°C, growth rates differed significantly among strains (one-way ANOVA, *F*_(17,72)_ = 33.02, *P* < 0.0001). When strains were grouped by genus, *Aspergillus* grew significantly faster than non-*Aspergillus* fungi (one-way ANOVA, *F*_(1,88)_ = 167.1, *P* < 0.0001). *Aspergillus* isolates consistently achieved higher maximum growth rates (4–6 mm/day) with broader optima around 20°C–25°C. In contrast, *Penicillium* isolates generally exhibited slower growth (2–4 mm/day) and narrower optima, with sharp declines in growth above 25°C–30°C. Other cheese-associated fungi (*Bifusarium domesticum* strain 554A and *Scopulariopsis* sp. strain 165_5) showed restricted growth across the thermal gradient, clustering at the lower performance range. These results indicate that *Aspergillus* species are more thermotolerant and capable of sustaining faster growth across a wide thermal range, whereas *Penicillium* and other cheese mold species are adapted to cooler conditions and exhibit narrower thermal niches.

To better understand the lower temperature limit of *A. westerdijkiae* growth, we quantified CFUs across a narrow temperature range between 9°C and 14°C (Fig. 4B). Fungal CFU counts differed significantly across temperatures (one-way ANOVA, *F*_5,24_ = 457, *P* < 0.0001). Growth was strongly suppressed at 10°C, with CFU counts close to no detection, but steadily increased with each incremental rise in temperature. A sharp transition in growth occurred between 10°C and 12°C, where populations expanded by several orders of magnitude, reaching densities of ~10⁶ CFU/mL at 13°C–14°C. This result suggests that while *A. westerdijkiae* (Aw2019) exhibits minimal growth at the lowest tested temperature, it may remain viable and capable of population expansion at near cheese cave temperature conditions. The performance shift around 10°C–11°C identifies this as a critical threshold for *A. westerdijkiae* cold tolerance.

To better understand how *A. westerdijkiae* may grow and compete with other microbes in a more realistic setting (on CCA medium), we also grew one strain of *A. westerdijkiae* (Aw2019) in co-culture with a desirable cheese rind fungus, *P. biforme* strain 162_3FA (Fig. 4C). Two-way ANOVA revealed significant effects of species identity (*F*_1,48_ = 1151.39, *P* < 0.0001), temperature (*F*_5,48_ = 499.99, *P* < 0.0001), and species × temperature interaction (*F*_5,48_ = 353.58, *P* < 0.0001) on fungal abundance. Tukey-adjusted post hoc comparisons revealed a temperature-dependent shift in competitive abilities between the two species. At 10°C, *Penicillium* was significantly more abundant than *A. westerdijkiae*. In contrast, *A. westerdijkiae* was significantly more abundant than *Penicillium* at all higher temperatures tested (Fig. 4C). These results suggest that temperature can mediate the competition of *A. westerdijkiae* with beneficial cheese rind fungi, and temperatures below 15°C are required to favor beneficial *Penicillium* molds over *A. westerdijkiae*.

### *A. westerdijkiae* is not normally found in the Northeastern United States based on global databases

The recent emergence of *A. westerdijkiae* in cheese production facilities in the United States could be due to this fungus being naturally present in habitats throughout the Northeastern United States. Recent events could have promoted the ability of this fungus to colonize cheese caves from natural environments and cause defects. Alternatively, the fungus could be absent from local fungal communities and may be introduced to cheese production facilities through materials used in cheese production or movement of fungal spores in human-built environments. To begin to consider potential sources of *A. westerdijkiae* at large spatial scales, we used data from GlobalFungi.com to determine the potential global distribution of this fungus. This database uses ITS metabarcoding studies to determine the presence of different fungal “species hypotheses” (73). We recognize that ITS is not the preferred marker for delineating different *Aspergillus* species (calmodulin, or CaM, is typically used), but past studies have demonstrated that (35) ITS sequences are divergent enough in section *Circumdati* to distinguish *A. westerdijkiae* from other closely related species. Additionally, an alignment of the ITS region of closely related species in section *Circumdati* demonstrates that there are several variable regions that distinguish strains of *A. westerdijkiae* from other *Aspergillus* species (Fig. S2).

**Fig 5:**
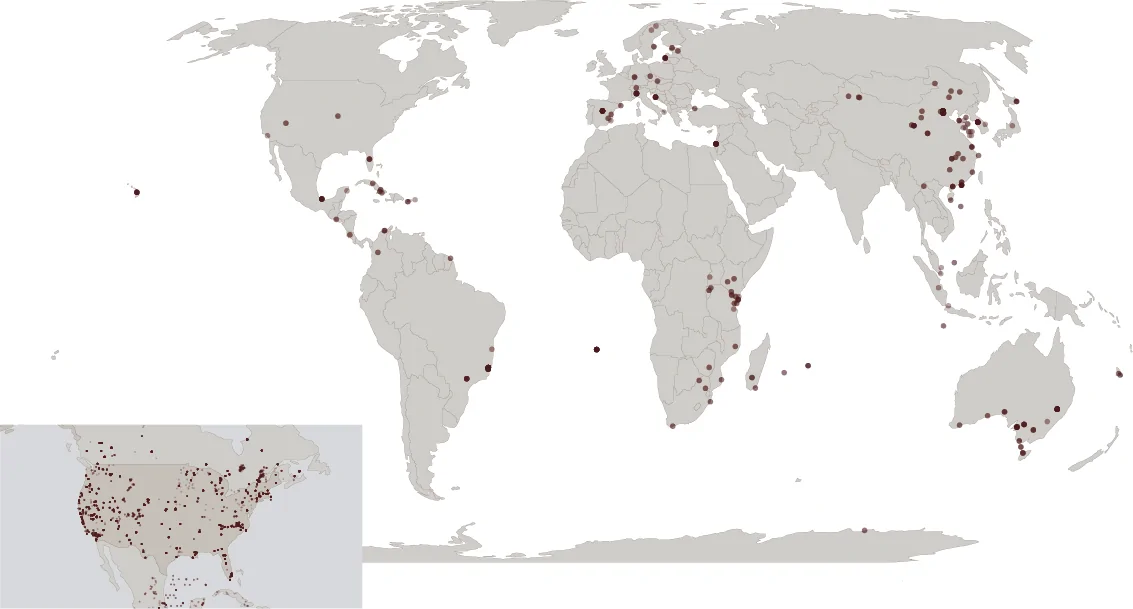
Global distribution of putative *A. westerdijkiae* ITS reads in the GlobalFungi database. In the larger global map, each dot represents a sample from a fungal biodiversity study, where ITS reads were assigned to the *A. westerdijkiae* species hypothesis from UNITE. Samples that had singletons (only one *A. westerdijkiae* read detected in the study) and where the relative abundance of *A. westerdijkiae* was less than 0.01% were excluded. Inset map shows locations of all studies included in GlobalFungi for North America, indicating that a lack of sampling is likely not a driver of lack of *A. westerdijkiae* ITS read detection in Eastern North America. Maps made with Geo Point Plotter.

When using several thresholds to remove potentially spurious or low-abundance samples (no singletons and only samples with greater than 0.01% relative abundance of *A. westerdijkiae*), mapping of putative *A. westerdijkiae* indicated that it has not been detected in field-collected samples from throughout the Northeastern United States (Fig. 5). In fact, occurrences of putative *A. westerdijkiae* ITS reads are rare across all of North America. One study detected putative *A. westerdijkiae* in a marine area near Venice, California, USA, and another study detected putative *A. westerdijkiae* in forest soil of Utah, USA. The lack of *A. westerdijkiae* in the Northeastern United States does not appear to be due to climate, as many studies have detected *A. westerdijkiae* across cooler regions of Europe, including a sample of litter from a Swedish forest where *A. westerdijkiae* was 3% relative abundance and mean annual temperature is 0.8°C (Table S6). The lack of detection is also not due to a lack of samples from the Northeastern United States in GlobalFungi, as there is good coverage of ITS sequencing studies across the Northeastern United States and Eastern Canada (inset of Fig. 5).

Studies where *A. westerdijkiae* reads were detected in GlobalFungi samples point to potential biogeographic and habitat preferences of this fungus in the wild. Putative ITS reads of *A. westerdijkiae* have been detected on all continents (with one sample from Antarctica), with Asia (38.1% of occurrences), Europe (13.4%), Africa (12.5%), Australia (12.5%), and South America (11.9%) having the most samples containing *A. westerdijkiae*. Of the 24 samples (7.5% of total occurrences) where putative *A. westerdijkiae* reads were detected in North America, most were in Mexico, Cuba, Hawaii, or other tropical regions. Outside of anthropogenic and cropland habitats, the most common natural habitats where putative *A. westerdijkiae* ITS reads have been detected across the globe include those associated with marine plants and sediments and/or mangrove swamps (19% of occurrences), woodland and forest soils and litter (56% of occurrences), and grasslands (11% of occurrences). Of the top 10 samples with the highest abundances of *A. westerdijkiae* ITS reads outside of anthropogenic or cropping systems, over half were marine habitats (Table S6), suggesting a tolerance or preference for salt by this fungus.

## DISCUSSION

Our comparative genomic analysis revealed distinct strains of *A. westerdijkiae* that contaminated different cheese-aging facilities in the Northeastern United States, which did not support our hypothesis of a single cheese-adapted clone spreading through the region. We are not aware of other studies that have attempted to delineate different strains of *A. westerdijkiae* using whole-genome sequences, and more genomes from strains from different locations and sources are needed to create a robust genomic framework for this important food spoilage mold, including population-level sampling to determine how much genomic diversity exists within *A. westerdijkiae* populations (we only sequenced a single *A. westerdijkiae* isolate per cheese production facility). Additional genomes of *A. westerdijkiae* from natural substrates and environments might help provide enough resolution to connect contaminating molds with their sources. Our genomic analysis also revealed that two different closely related *Aspergillus* species—both *A. westerdijkiae* and *A. ostianus—*were causing rind development issues at one facility. We are only aware of one other report of *A. ostianus* contaminating cheeses (63), but this fungus may need to be on the radar of artisan cheese producers.

The European Union has set regulatory limits for OTA in foods that range from 0.5 to 80 µg/kg depending on the product, while the U.S. Food and Drug Administration only has a non-enforceable “referral level” of 20 µg/kg that is used to monitor foods with potential OTA risks (74). Several studies have reported OTA concentrations in cheese well above these levels, including a study in Brazil that reported OTA in *Aspergillus*-contaminated artisanal cheeses, with concentrations ranging from 1 to >1,000 µg/kg (63). Studies in European cheeses report generally low to moderate contamination levels (1.3–22.4 µg/kg) (75). Beyond cheese systems, *A. westerdijkiae* has been shown to produce OTA in other foods such as salami, with concentrations reaching 21,000 µg/kg under optimal environmental conditions (76). Compared to these reports, the levels of OTA observed in our experimental lab conditions, particularly for strain Aw2012, are notably elevated, suggesting that certain strains may possess higher toxigenic potential under favorable conditions. It is important to note that we did not have an opportunity to test OTA in the actual cheese products that were contaminated, and instead used a small volume (20 mL) of cheese curd in a Petri dish to assess OTA production. This might increase total OTA detected because of the high surface-to-volume ratio. We were unable to measure OTA production on actual cheeses during a contamination event because we were only sent small samples of cheese for fungal isolations, and cheeses that were contaminated were discarded to avoid further contamination of other cheeses. Future work is needed to directly measure OTA production in artisan cheeses made in the Northeastern United States during an *A. westerdijkiae* contamination event to fully understand the safety risks of this mold in artisan cheese production. Given that we observed some *A. westerdijkiae* strains with low levels of OTA production, not all contamination events may pose safety risks, and cheesemakers should use direct OTA testing of their products to evaluate potential risks.

The upregulation of several pathways in our RNA-seq data suggests that *A. westerdijkiae* alters its metabolism to cope with the challenging nutritional conditions of the cheese rind substrate. While this fungus is often found on plant and soil substrates, it appears to be able to easily shift global gene expression patterns to thrive on the unique substrate that is cheese. Carbohydrate metabolism was one KEGG module with a high number of enriched metabolic pathways, and this transcriptomic response may be due to carbohydrate availability in cheese. When cow’s milk is first enzymatically digested by lactic acid bacteria at the outset of the cheese production process, many of the freely available carbohydrates are digested from the substrate (40, 41). Thus, when microbes like *A. westerdijkiae* colonize a cheese rind, they must catabolize lactate or break down any residual lactose (77), which might explain the observed changes in expression of genes in these pathways. Amino acid and lipid metabolism pathways were also enriched in the upregulated gene set and align with what is known about how fungi can transform proteins and fats in cheese to access resources for growth (43, 77).

Temperature strongly influenced the growth dynamics of both typical cheese rind filamentous fungi and the spoilage *Aspergillus* species, revealing distinct thermal niches across species in this study (Fig. 4A). In cheese environments, these differences suggest that *Aspergillus* species may hold a competitive advantage under warmer conditions, but such an advantage is lost at colder temperatures where *Penicillium* species dominate (Fig. 4C). Previous studies have shown that temperature modulates a variety of fungal processes, from spore germination and hyphal extension to secondary metabolism and enzyme secretion, thereby influencing growth rates and competitive ability (78–82). The ability of *Aspergillus* isolates to sustain faster growth at higher temperatures (Fig. 4A) could enable them to colonize cheese rinds more aggressively, potentially displacing *Penicillium* species that are traditionally central to the development of desirable rind textures and flavors.

Our results demonstrate that *Aspergillus westerdijkiae* can sustain slow but measurable growth at low temperatures, with CFU counts increasing between 11°C and 14°C but remaining strongly suppressed at 10°C (Fig. 4B). This suggests that while 10°C approaches the lower critical thermal limit for this species, an increase above this threshold may permit gradual proliferation. The persistence of *A. westerdijkiae* at 11°C–14°C indicates that standard cave conditions may not be fully effective at excluding this species, and the need for cheese producers to carefully manage temperatures in cheese aging facilities. It is important to note that our experiments were done in Petri dishes, and sometimes on a medium (PDA) with a very different composition than cheese, and specific temperature thresholds for managing *A. westerdijkiae* may depend on the specific cheese production environment. But follow-up conversations with some of the producers affected by the *A. westerdijkiae* strains characterized in this study indicated that careful and consistent control of cave temperatures below 12°C allowed them to effectively eliminate the negative effects of this fungus in their caves. Collectively, our experiments and these observations demonstrate that small shifts in cheese aging facility temperatures, whether driven by climate change, energy-saving practices, or equipment failure, could alter the competitive balance between *Aspergillus* and desirable cheese rind fungi.

One important question about the ecology of *A. westerdijkiae* and other spoilage *Aspergillus* species in artisan cheese in the Northeastern United States is why have these fungi colonized multiple independent production facilities over the past decade? Where do they come from, and what conditions are required for successful invasion into these caves? A range of materials used in cheese production, from raw milk to the wooden boards used for aging, can be the source of microbial cells that contribute to cheese rind communities (7). *A. westerdijkiae* has been reported to contaminate cheeses made in Europe and South America (23, 63, 83), but historically this fungus has not been known to be problematic for cheese production in the United States. Our work exploring the global distribution of this fungus suggests that it is not a typical species found in natural areas of Eastern North America, although metabarcoding databases have limited geographic breadth, and additional field surveys are needed to determine whether *A. westerdijkiae* can be found in natural areas of the Northeastern United States. The data from GlobalFungi often indicated that *A. westerdijkiae* was associated with marine environments. One possible explanation that needs further exploration is that the emergence of *A. westerdijkiae* in these cheese facilities is through the use of minimally processed sea salts. Salts can contain viable spores of *Aspergillus*, *Penicillium*, and other fungal species (84). We are unaware of studies demonstrating the presence of *A. westerdijkiae* in sea salts, but this may be a fruitful area of future research for understanding how these spoilage molds may spread around the world and into food production environments.

## Data Availability

Raw Illumina fastq reads from each of these genomes have been deposited in the National Center for Biotechnology Information (NCBI) SRA (BioProject # PRJNA1171465). Raw Illumina fastq reads from the RNA-seq experiments have been deposited in the NCBI SRA (BioProject # PRJNA1171471).

## SUPPLEMENTAL MATERIAL

Supplemental figures aem.00032-26-s0001.docx Fig. S1 and S2.

Table S1 aem.00032-26-s0002.xlsx Genomes and strains used in this study.

Table S2 aem.00032-26-s0003.xlsx Number of unique genes identified in different *A. westerdijkiae* strains and KOBAS-i pathway enrichment results.

Table S3 aem.00032-26-s0004.xlsx Detailed antiSMASH outputs for cheese-associated *Aspergillus* species.

Table S4 aem.00032-26-s0005.xlsx Expression values for all genes in *A. westerdijkiae* strain Aw2019, including genes that were differentially expressed.

Table S5 aem.00032-26-s0006.xlsx KOBAS-i pathway enrichment outputs of upregulated and downregulated genes.

Table S6 aem.00032-26-s0007.xlsx Occurrences of putative *A. westerdijkiae* in the GlobalFungi database.

ASM does not own the copyrights to Supplemental Material that may be linked to, or accessed through, an article. The authors have granted ASM a non-exclusive, world-wide license to publish the Supplemental Material files. Please contact the corresponding author directly for reuse.
