# Supplementary material for: Genomic diversity and thermal niches of Aspergillus molds disrupting rind formation of surface-ripened cheeses
Source: Appl Environ Microbiol. 2026 Sep 8;92(10):e00032-26. doi: 10.1128/aem.00032-26 (PMC13689457; doi:10.1128/aem.00032-26)
Supplement: Supplementary file 1 [file aem.00032-26-s0001.docx]

**Figure S1:** Growth rate of one *Aspergillus westerdijkiae* strain (Aw2019) on cheese curd agar at different temperatures. Growth rate was measured in the same way as in Figure 4. Points represent the mean of five replicates and error bars are one standard deviation of the mean.

**Figure S2:** Alignment of the ITS region of *Aspergillus westerdijkiae* and closely related species in section [*Circumdati*](http://paperpile.com/b/rBC7yA/ccvTN). Zoomed in views show variable regions that distinguish *Aspergillus* *westerdijkiae* ITS sequences from other species.
